# The association between childbirth-related fear, childbirth readiness, and fertility intentions, and childbirth readiness as the mediator

**DOI:** 10.1186/s12978-023-01607-x

**Published:** 2023-04-21

**Authors:** Tieying Zeng, Bingbing Li, Ke Zhang, Ye Chen, Mengmei Yuan, Meiliyang Wu, Huimin Zhao, Zining Zhu, Dandan Ju

**Affiliations:** 1 Department of Nursing, Tongji Hospital, Tongji Medical College, Huazhong University of Science and Technology, 1095 Jiefang Avenue, Wuhan, 430030 China; 2 School of Nursing, Tongji Medical College, Huazhong University of Science and Technology, 13 Hangkong Road, Wuhan, 430030 China

**Keywords:** Childbirth-related fear, Fertility intentions, Childbirth readiness, Social support, Mediator, China

## Abstract

**Background:**

Fertility intentions have been proved to be a reliable predictor of actual fertility behaviour. Also, childbirth-related fear (CBRF) has been proven to be negatively associated with childbirth readiness and fertility intentions among women, while childbirth readiness was positively related to fertility intentions. However, the associations and potential mechanisms between CBRF, childbirth readiness, and fertility intentions remain unknown. This study aimed to investigate the unique association between CBRF, childbirth readiness, and fertility intentions and whether childbirth readiness would mediate the relationship between CBRF and fertility intentions.

**Method:**

A cross-sectional study of women (N = 1119, aged 16–53 years) who gave birth within 72 h was conducted. Using a convenience sampling, women were recruited from obstetric wards—10 comprehensive hospitals and 3 specialized hospitals in 7 provinces in mainland China. Pearson correlation was used to examine the relationship between CBRF, childbirth readiness, fertility intentions, and social support. Multivariate linear regression was further used to analyze the association between demographic and personal characteristics, CBRF, childbirth readiness, and fertility intentions. Mediation analysis was used to examine whether childbirth readiness mediates the relationship between CBRF and fertility intentions.

**Results:**

Women with high childbirth readiness (*β* = 0.09, *P* = 0.002) had higher fertility intentions. However, women with high CBRF (*β* = − 0.17, *P* < 0.001) were more likely to have lower fertility intentions. CBRF had both direct and indirect effects on the level of fertility intentions. As predicted, childbirth readiness mediated the relationship between CBRF and the level of fertility intentions (estimate = − 0.012, 95% bootstrap CI: − 0.021 to − 0.005). Higher CBRF was associated with lower scores of childbirth readiness, which was associated with lower levels of fertility intentions.

**Conclusions:**

This study established the evidence that CBRF had both direct and indirect effects on the level of fertility intentions and childbirth readiness mediated the relationship between CBRF and the level of fertility intentions. Specifically, higher CBRF was associated with lower scores of childbirth readiness, which was associated with lower levels of fertility intentions. This finding suggested that it is important for health policymakers and health providers to pay more attention to improving women’s childbirth readiness, which might reduce the negative influence of CBRF on fertility intentions, thus strengthening their fertility intentions.

## Plain language summary

Over the past two decades, fertility rates have been dropping steadily in most countries. Fertility intentions have been proven to be a reliable predictor of actual fertility behaviour. The worldwide sluggish fertility trend has brought about a series of issues, such as accelerated population ageing, shrinking workforce, and economic decline. China has the largest national population in the world, accounting for nearly one-fifth of the world's population, thus having a huge impact on global population trends. Therefore, in the context of significant fertility decline and population policy changes, timely research on fertility intention is of great significance for China and other low-fertility countries. CBRF has been proven to be negatively associated with childbirth readiness and fertility intentions among women, while childbirth readiness was positively related to fertility intentions. This study aimed to investigate the unique association between fertility intentions, CBRF, and childbirth readiness, and whether childbirth readiness would mediate the relationship between CBRF and fertility intentions. In this cross-sectional study, we found that women with high childbirth readiness had higher fertility intentions. However, women with high CBRF were more likely to have lower fertility intentions. Furthermore, CBRF had both direct and indirect effects on the level of fertility intentions. Childbirth readiness mediated the relationship between CBRF and the level of fertility intentions. Higher CBRF was associated with lower scores of childbirth readiness, which was associated with lower levels of fertility intentions. This finding suggested that it is important for health policymakers and health providers to pay more attention to improving women’s childbirth readiness, which might reduce the negative influence of CBRF on fertility intentions, thus strengthening their fertility intentions.

## Introduction

Over the past two decades, fertility rates have been dropping steadily in most countries. Research has found that 179 of 204 countries saw a decline in the total fertility rate (TFR) in the last ten years [1]. Half of these 204 countries reached below-replacement-level TFR by 2019, with one-fifth of them reaching an ultra-low TFR of 1.5 or lower. The worldwide sluggish fertility trend has brought about a series of issues, such as accelerated population ageing, shrinking workforce, and economic decline [2]. China has the largest national population in the world, accounting for nearly one-fifth of the world's population, thus having a huge impact on global population trends [3]. In 1979, the government of China established the one-child policy for families, and since the beginning of the policy, the TFR has decreased and the challenges described above have emerged [4]. Therefore, with the aim of reliving the declining fertility rates, improving the population structure, and actively responding to the ageing population, China has been gradually reforming the fertility policies. This is through implementing the “selective two-child policy” and “universal two-child policy” in 2013 and 2015 [5], respectively. Although, there was a brief baby booming—a rise in the number of births from 16.55 million in 2015 to 17.86 million in 2016, the number declined constantly to 12.00 million by 2020 [6]. To raise the numbers, China launched the three-child policy in 2021 as a supportive measure [7], allowing all couples to have up to three children.

Fertility intentions have been regarded as a reliable predictor of actual fertility behavior [8]. Although there may be discrepancies and complex associations between fertility intentions and actual fertility, previous studies have reported that fertility intentions have a positive and independent effect on actual fertility [9, 10]. Therefore, in the context of significant fertility decline and population policy changes, timely research on fertility intention is of great significance for China and other low-fertility countries. Previous studies concerning fertility intentions among Chinese women have mainly focused on the influence of economic resources, fertility policies, and culture formation [11–13]. Factors stemming from women themselves have been ignored. In this study, fertility intentions refer to wanting to have another child after experiencing the most recent birth, which could be to a large extent influenced by the experience of the recent birth and pregnancy process. Some studies have found that childbirth-related fear (CBRF) as the real feeling and expectation of fear and anxiety concerning giving birth among women, might be the main negative influencing factor of fertility intentions [14–16]. CBRF includes fear of the unknown, the potential for injury, the inability to cope with labor pain, not having the capacity to give birth, losing control, and inadequate support from care providers [14, 17]. It is an obstacle for pregnant women to overcome and could lead to decreased abilities in coping with birth, lower birth satisfaction, and can be detrimental to women’s physical and psychological health. As a result, high levels of CBRF might reduce women’s fertility intentions due to negative consequences and experiences brought by it [16]. Therefore, we propose that CBRF negatively relates to fertility intentions.

Childbirth readiness could cause lesser maternal complications and improve women’s childbirth experiences [18, 19], thus potentially strengthening their fertility intentions [20, 21]. Childbirth readiness could also reflect women’s birth preparedness in terms of their knowledge, psychological aspect, and planning [22]. That is, women who are prepared for childbirth have improved birth confidence in the knowledge and information acquired, which could help them positively cope with difficulties encountered during pregnancy and the birth process [23, 24]. Previous studies have proved that having less CBRF is related to having adequate childbirth readiness and positive birth experiences [21, 25]. In this regard, women with less CBRF are able to keep a positive mindset and focus on childbirth preparations. The reduction of CBRF as well as the improvement of childbirth readiness could strengthen women’s self-confidence for future pregnancies and birth, thus contributing to their fertility intentions [23].

Based on the evidence, we hypothesize that CBRF negatively associates with childbirth readiness and fertility intentions. Considering that childbirth readiness is positively associated with fertility intentions, we further hypothesize that childbirth readiness mediates the relationship between CBRF and fertility intentions. To our knowledge, no study to date has examined the relationship between fertility intentions, CBRF, and childbirth readiness, and explored if childbirth readiness is a mediating factor. We therefore, aimed to assess such association in this study.

## Methods

### Study design

A cross-sectional study of women (N = 1119) in obstetric wards—10 comprehensive hospitals and 3 specialized hospitals from 7 provinces/municipalities/autonomous regions in mainland China. The National Bureau of Statistics of China divided Chinese economic regions into four regions. Based on the four regions, we randomly selected seven provinces/municipalities/autonomous regions. Then, we contacted the tertiary hospitals and secondary hospitals in the seven provinces/municipalities/autonomous regions that were sampled. This study was conducted from November 2021 to March 2022.

### Participants

A convenient sampling method was used to recruit participants in this study. We included women: (1) who gave birth within 72 h; and (2) were willing to participate in the study. Women with: (1) psychological diseases (e.g., diagnosis of depression, bipolar disorder, post-traumatic stress disorder, etc.); and (2) severe obstetric complications, were excluded from the study. In the pilot study, the standard deviation (SD) of fertility intention scores was 1.50 in some of the hospitals where we conducted the survey; with a maximal tolerance of 0.10 and error of type I at 5%, the minimal sample sizes were 865. Considering 10% invalid sample size, at least 951 participants were needed. Finally, a total of 1119 participants who met our inclusion criteria were recruited.

### Ethical considerations

The study protocol was approved by the Ethics Committee of Tongji Hospital, Tongji Medical College, Huazhong University of Science and Technology (reference number: TJ-IRB20210755). All participants completed the written informed consent before participating in the study.

## Measures

### Data collection

A self-administered online questionnaire was used to collect data. In the process of the questionnaire survey, trained nurses in the ward distributed electronic questionnaires to participants one-on-one.

### Demographic variables

The demographics included age, education level, marital status, marital satisfaction (satisfied/unsatisfied), residence area (urban/rural), monthly household income, pregnancy sleep status, pregnancy exercise status, parity (nulliparous/ multiparous), adverse obstetric history, self-reported obstetric complications, pregnancy life events, etc. The following variables were classified as: age (years)—less than 26, 26–35, and more than 35; education level—primary school or lower, junior high school, senior high school, and college or higher; monthly household income—less than 3001¥, 3001–5000¥, 5001–10,000¥, and more than 10,000¥; pregnancy sleep status—well, moderate, and poor; pregnancy exercise status—always, often, occasionally, and never; with adverse obstetric history, self-reported obstetric complications, and pregnancy life events categorized as yes or no.

### Social support

Social support was measured using the Chinese version of the medical outcomes study social support survey (MOS-SSS), which is a 20-item scale. The MOS-SSS scale includes one item evaluating the support network and 19 items assessing the availability of social support in four dimensions—emotional, tangible, affectionate, and positive social interaction. The MOS-SSS is measured on a 5-point Likert scale ranging from 20 to 100, with a higher score indicating greater social support. The Cronbach α and 2-week test–retest reliability for the Chinese version of the MOS-SSS were 0.98 and 0.84, respectively [26]. Cronbach α for the present study was 0.97.

### Childbirth-related fear (CBRF)

The Chinese version of the fear of childbirth scale (FOBS) was used to estimate the CBRF [27]. The FOBS is a 2-item visual analogue scale, with the question: “how did you feel about the approaching birth in your recent pregnancy?” Two separate items were used to assess the degree of worry and fear, both of which were indicated on a scale of 0 to 10. The total score of the FOBS was calculated as the mean value of the two items. A higher score indicates a higher level of CBRF. The initial Chinese FOBS demonstrated a strong internal consistency, with a Cronbach α of 0.91 [28]. In the present study, the FOBS reported a Cronbach α of 0.89.

### Childbirth readiness

Childbirth readiness was assessed using the Chinese version of the childbirth readiness scale (CRS)[22]. The CRS is an 18-item questionnaire with four dimensions—self-management, information literacy, birth confidence, and birth plan, assessed on a 5-point Likert scale ranging from 1 (strongly disagree) to 5 (strongly agree). A higher score indicates greater childbirth readiness. The CRS has been validated in Chinese pregnant women, with good reliability (Cronbach α = 0.94 and split-half reliability = 0.88) [22]. In the present study, the CRS Cronbach α was 0.96.

### Fertility intentions

Individuals’ fertility intentions were evaluated using the following question: “Would you refuse to have another child due to the experience of this birth?” The five possible responses were: “definitely not”, “probably not”, “not sure”, “probably yes”, and “definitely yes”, on a scale of 1 to 5 [29]. In this study, reverse scoring was used to calculate the values, with higher scores indicating higher levels of fertility intentions.

### Data analyses

IBM SPSS v26.0 (SPSS Inc., Chicago, IL, USA) and PROCESS 4.0 for SPSS macro-program [30] were used to conduct data analysis. All continuous variables were tested for normality, and achieved normality. Independent *t*-test and one-way ANOVA were used to describe the differences in demographic and personal characteristics with fertility intentions. Pearson correlation was used to examine the relationship between fertility intentions, CBRF, childbirth readiness, and social support. Multivariate linear regression was further used to analyze the association between demographic and personal characteristics (all variables shown in Table 1), fertility intentions, CBRF, childbirth readiness, and social support. Two-tailed *P* < 0.05 was set as the significant level. The mediation model tested whether childbirth readiness mediated the relationship between CBRF and fertility intentions. The 5000 bootstrapped samples based on bias-corrected confidence intervals were used to examine the indirect effect. If the 95% bootstrap confidence interval does not include zero, the effect was regarded as significant.

**Table 1:** Demographic and personal characteristics of participants by fertility intentions (N = 1119)

Variables	N (%)	Fertility intentions		
		Mean (SD)	*t/F*	*P* value
Age (years)			0.35	0.707
≤ 25	161 (14.39)	3.52 (1.32)		
26–35	865 (77.30)	3.50 (1.28)		
≥ 36	93 (8.31)	3.39 (1.46)		
Education level			1.83	0.140
Primary school or lower	21 (1.88)	4.05 (1.53)		
Junior high school	161 (14.39)	3.39 (1.39)		
Senior high school	166 (14.83)	3.57 (1.30)		
College or higher	771 (68.90)	3.48 (1.27)		
Marital status			-0.53	0.597
Married	1079 (96.43)	3.49 (1.30)		
Others (single/divorced/widowed)	40 (3.57)	3.60 (1.34)		
Marital satisfaction			2.71	0.007
Satisfied	1088 (97.23)	3.51 (1.29)		
Unsatisfied	31 (2.77)	2.87 (1.48)		
Residence area			-0.62	0.534
Urban area	880 (78.64)	3.48 (1.29)		
Rural area	239 (21.36)	3.54 (1.34)		
Monthly household income (¥)			1.71	0.163
≤ 3000	253 (22.61)	3.59 (1.31)		
3001–5000	357 (31.90)	3.41 (1.34)		
5001–10,000	340 (30.38)	3.44 (1.29)		
> 10,000	169 (15.10)	3.63 (1.20)		
Pregnancy sleep status			29.34	< 0.001
Well	636 (56.84)	3.72 (1.26)		
Moderate	440 (39.32)	3.25 (1.26)		
Poor	43 (3.84)	2.58 (1.40)		
Pregnancy exercise status			14.01	< 0.001
Always	129 (11.53)	4.12(1.29)		
Often	424 (37.89)	3.51 (1.29)		
Occasionally	533 (47.63)	3.36 (1.26)		
Never	33 (2.95)	2.97 (1.36)		
Parity			0.519	0.604
Nulliparous	758 (67.74)	3.51 (1.24)		
Multiparous	361 (32.26)	3.46 (1.42)		
Adverse obstetric history			2.01	0.157
Yes	99 (8.85)	3.53 (1.40)		
No	1020 (91.15)	3.49 (1.29)		
Self-reported obstetric complications			1.07	0.285
Yes	125 (11.17)	3.38 (1.25)		
No	994 (88.83)	3.51 (1.31)		
Pregnancy life events			1.44	0.149
Yes	28 (2.50)	3.14 (1.18)		
No	1091 (97.50)	3.50 (1.30)		

Nulliparous: women who have never given birth; Multiparous: women who have given birth one or more times

*SD* standard deviation

## Results

### Participant demographics

Participants’ mean age was 29.53 (Standard Deviation = 4.33, Range = 16–53) years. More detailed characteristics of the participants are presented in Table 1. Significant differences were seen between variables of pregnancy sleep status, pregnancy exercise status, and social support with fertility intentions (all *P* ≤ 0.007).

### Correlation analyses

Table 2 reports the mean scores and correlation between fertility intentions, CBRF, childbirth readiness, and social support. The level of fertility intentions was negatively associated with CBRF (*r* = − 0.237, *P* < 0.001) and positively related to childbirth readiness (*r* = 0.180, *P* < 0.001) and social support (*r* = 0.185, *P* < 0.001). CBRF was negatively related to childbirth readiness (*r* = − 0.148, *P* < 0.001) and social support (*r* = − 0.117, *P* < 0.001). Childbirth readiness was positively correlated with social support (*r* = 0.246, *P* < 0.001). All *P* values were significant and the effect sizes were more than 0.1 between the variables.

**Table 2:** Mean, standard deviations, and correlations of variables

Variables	Mean	SD	1	2	3	4
1. Fertility intentions	3.49	1.30	1	− 0.237**	0.180**	0.185**
2. CBRF	4.91	2.48		1	− 0.148**	− 0.117**
3. Childbirth readiness levels	77.39	10.60			1	0.246**
4. Social support	72.86	14.72				1

*CBRF* childbirth-related fear, *SD* standard deviation; ***p < 0.001

### Associated factors for fertility intentions

The multivariate linear regression analysis in this study revealed that compared to women with a well pregnancy sleeping pattern, those with moderate (*β* = − 0.10, *P* = 0.001) and poor (*β* = − 0.12, *P* < 0.001) sleeping patterns, had lower fertility intentions (Table 3). Women who reported often (*β* = − 0.14,* P* = 0.004), occasionally (*β* = − 0.15,* P* = 0.002), and never (*β* = − 0.09, *P* = 0.006) in pregnancy exercise status had a lower level of fertility intentions than those reporting always in exercise status. Those having higher social support (*β* = 0.11, *P* < 0.001) and childbirth readiness (*β* = 0.09, *P* = 0.002) had a higher level of fertility intentions. Whereas, women with higher CBRF (*β* = − 0.17,* P* < 0.001) were more likely to be in a lower level of fertility intentions.

**Table 3:** Multivariate linear regression analysis of the associations between variables and fertility intentions (N = 1119)

		Fertility intentions			
Variables	B	*β*	95%*β* Lower	95%*β* Upper	*P* value
CBRF	− 0.09	− 0.17	− 0.12	− 0.06	< 0.001
Childbirth readiness levels	0.01	0.09	0.01	0.02	0.002
Social support	0.01	0.11	0.01	0.02	< 0.001
Pregnancy sleep status					
Well (reference)					
Moderate	− 0.26	− 0.10	− 0.41	− 0.10	0.001
Poor	− 0.79	− 0.12	− 1.18	− 0.40	< 0.001
Pregnancy exercise status					
Always (reference)					
Often	− 0.36	− 0.14	− 0.61	− 0.11	0.004
Occasionally	− 0.40	− 0.15	− 0.65	− 0.15	0.002
Never	− 0.68	− 0.09	− 1.15	− 0.20	0.006

Only variables with* P* < 0.05 were showed; *CBRF* childbirth-related fear; *β* = standardized coefficient

Adjusted* R*^*2*^ = 0.125

### Childbirth readiness as a mediator for CBRF and fertility intentions

**Fig. 1:**
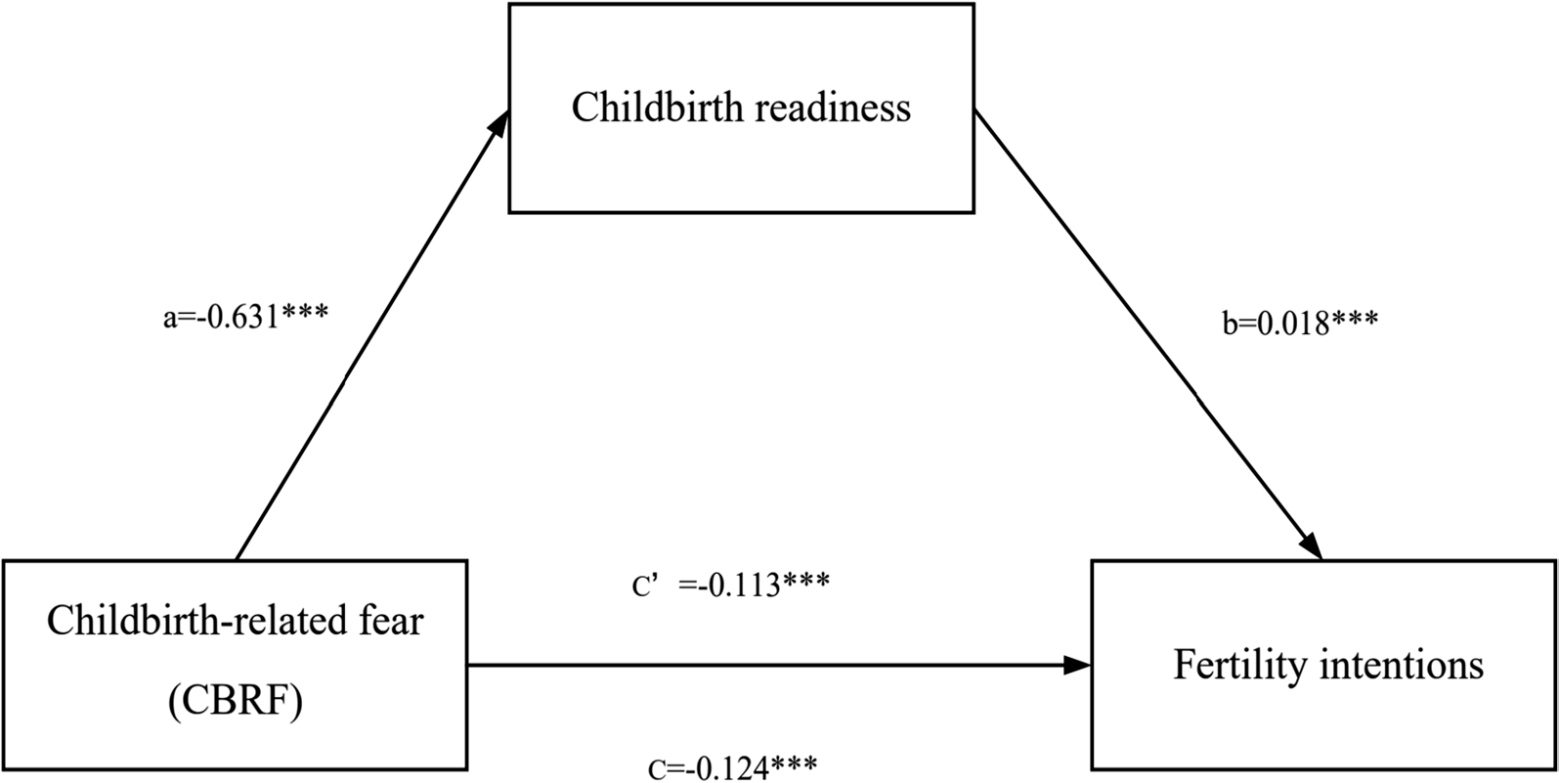
Mediating role of childbirth readiness in the relationship between CBRF and fertility intentions. ***p < 0.001

The total, direct, and indirect effects of the mediation model are reported in Table 4. CBRF on fertility intentions remained significant after introducing childbirth readiness into the model. The mediation analysis showed, childbirth readiness was negatively associated with CBRF (a = − 0.631, 95% *CI*: − 0.879 to − 0.384), and positively related to fertility intentions (b = 0.018, 95% *CI*: 0.011 to 0.025). CBRF was further negatively related to fertility intentions (c’ = − 0.113, 95% *CI*: − 0.143 to − 0.083). The mediation analysis supported our study hypothesis of childbirth readiness having an indirect effect on the relationship between CBRF and fertility intentions (ab = − 0.012, 95% bootstrap *CI*: − 0.021 to − 0.005). Figure 1 further illustrates the mediation effects.

**Table 4:** CBRF as the predictor of fertility intentions, mediated by childbirth readiness levels (N = 1119)

Model pathways	Estimates	Boot *SE*	95% *CI* (bootstrapping = 5000)
Total effect CBRF → Y(c)	− 0.124	0.015	− 0.154 to − 0.094
Direct effect CBRF → Y(c’)	− 0.113	0.015	− 0.143 to − 0.083
Indirect effect CBRF → M → Y(a × b)	− 0.012	0.004	− 0.021 to − 0.005

Y = level of fertility intentions; M = childbirth readiness levels; *CI* confidence interval; *SE* standard error; *CBRF* childbirth-related fear

## Discussion

This is the first study to assess the association between CBRF, childbirth readiness, and fertility intentions, with childbirth readiness as the mediating factor in China, Asia, and globally. We found CBRF to be a negative predictor, while childbirth readiness was a positive predictor for fertility intentions. The mediation model demonstrated childbirth readiness mediated the relationship between CBRF and fertility intentions, which supported our hypothesis. Specifically, higher CBRF was associated with lower childbirth readiness, which was associated with lower fertility intentions. The current study provides evidence of potential mechanisms between the association of CBRF, childbirth readiness, and fertility intentions.

In the present study, we found that women with higher levels of CBRF were more likely to be in lower levels of fertility intentions. This is consistent with previous results suggesting that the development of fear of childbirth is a vicious circle of negative experiences of childbirth from present pregnancy influencing future pregnancy [31]. High levels of CBRF have been regarded to associate with passive mental symptoms, negative birth experiences, and fear of future pregnancy, which probably lead to women’s low levels of fertility intentions [16, 32, 33]. In addition, we found that women having higher childbirth readiness had higher levels of fertility intentions. Similarly, a previous qualitative study exploring women’s perceptions of childbirth experiences found that childbirth preparation could improve women’s self-confidence for a future birth [18]. Women with well preparedness for childbirth could understand the labour process and overcome fears and worries about labour, which could help them achieve a positive labour experience, contributing to fertility intentions [34].

In the current mediation model, we found that childbirth readiness partially mediated the relationship between CBRF and the level of fertility intentions. The specific relation is that higher CBRF was associated with lower scores on childbirth readiness, which was associated with a lower level of fertility intentions. Previous studies have provided some clues for links between CBRF, childbirth readiness, and fertility intentions. A study conducted among 204 primiparous pregnant women in Iran found that women who regularly attended childbirth preparation classes had lower scores in fear of childbirth compared with non-attending ones [25]. In a qualitative study, pregnant women obtaining the pre-birth training acknowledged that the knowledge and skills in health management had improved their childbirth experience; they developed more confidence, while worries and concerns about childbirth had been reduced in this process [24]. Another qualitative study found that women who experienced a greater preparedness during pregnancy had a manageable childbirth fear and positive birth experience, which strengthened their self-confidence for a future birth [23]. High childbirth readiness was meaning to be well prepared in self-management, information literacy, birth confidence, and birth plan, which was associated with reduced childbirth fear and improved birth experience, benefiting women’s fertility intentions [22]. Although these studies that aimed at promoting pregnant women’s childbirth readiness have reduced women’s childbirth fear and improved their childbirth experience and self-confidence for a future birth, the specific association among childbirth fear, childbirth readiness, and fertility intentions was not clear. In the present study, we first found that childbirth readiness partially mediated the relationship between CBRF and the level of fertility intentions. This study revealed the possible mechanism that underly the relation between CBRF, childbirth readiness, and fertility intentions. The results suggest that interventions enhancing women’s childbirth readiness have the promise to reduce the negative influence of CBRF and improve their fertility intentions.

In this study, several other factors were related to women’s fertility intentions. We found that compared to women with a well sleeping pattern during pregnancy, women with moderate and poor sleeping patterns had lower fertility intentions. Our study supports the evidence that poor sleeping patterns during pregnancy may account for adverse pregnancy outcomes and negative pregnant experiences [35, 36], which may have negative influences on women’s fertility intentions. Likewise, women who often, occasionally or never exercise during pregnancy have lower fertility intentions than those who always exercise. That is, exercising daily during pregnancy is beneficial for women as it reduces the risk of pregnancy-related complications, and promotes a greater sense of well-being [37, 38]. Future studies should consider investigating the link between sleep and exercise as motivating factors for fertility intentions. We found women with higher social support had high fertility intentions. Our findings are consistent with previous studies which found that social support from family members including partners, parents, and parents-in-law increases women’s fertility intentions [29, 39, 40]. For Chinese couples, their parents are major sources providing child-bearing support, which could be helpful in increasing their fertility intentions [40]. With the reform of the social economic system and ideology, women in China have assumed more and more social roles. The gender role has changed qualitatively from "men taking charge of the outside and women taking charge of the inside" to "men and women sharing responsibility". Currently, the majority of women in China are engaged in full-time employment, which is hard for them to meet the demands imposed by their dual roles as workers and mothers [41]. In this background, support from the husband might be essential for them to decide whether have another child. Social support such as emotional support, tangible help, validation and acceptance, and appraisal support is beneficial for pregnant women to maintain health and well-being. In addition, adequate social support can assist in meeting pregnant women’s emotional needs, improving their self-esteem and coping abilities [42]. All of those could help women to experience a positive pregnancy and childbirth and influence their fertility intentions.

First, in this cross-sectional study, the causal directions of the association cannot be ascertained. Longitudinal studies need to explore how the three variables function in time. Second, the information of CBRF and childbirth readiness was collected within 72 h after giving birth, so recall bias may exist. Future studies could survey women’s CBRF and childbirth readiness during their pregnancy. Third, women’s fertility intention was measured using a single question. In follow-up studies, scales or other more standardized tools could be used to evaluate fertility intentions and the results could be compared.

## Conclusions

This study established the evidence that CBRF had both direct and indirect effects on the level of fertility intentions and childbirth readiness mediated the relationship between CBRF and the level of fertility intentions. Specifically, higher CBRF was associated with lower scores of childbirth readiness, which was associated with lower levels of fertility intentions. This finding suggested that it is important for health policymakers and health providers to pay more attention to improving women’s childbirth readiness, which might reduce the negative influence of CBRF on fertility intentions, thus strengthening their fertility intentions.

## Data Availability

The data used in the current study are available from the corresponding author upon reasonable request.

## Abbreviations

CBRF: Childbirth-related fear
MOS-SSS: The medical outcomes study social support survey
FOBS: The fear of childbirth scale
CRS: The childbirth readiness scale
